# Examination of Potential of Thermopile-Based Contactless Respiratory Gating

**DOI:** 10.3390/s21165525

**Published:** 2021-08-17

**Authors:** Qi Zhan, Wenjin Wang, Xiaorong Ding

**Affiliations:** 1 College of Electrical and Information Engineering, Hunan University, Changsha 410082, China; zhanqi@hnu.edu.cn; 2 Department of Electrical Engineering, Eindhoven University of Technology, 5612 AZ Eindhoven, The Netherlands; 3 School of Life Science and Technology, University of Electronic Science and Technology of China, Chengdu 611731, China

**Keywords:** thermopile array, thermal imaging, respiratory rate, remote screening

## Abstract

To control the spread of coronavirus disease 2019 (COVID-19), it is effective to perform a fast screening of the respiratory rate of the subject at the gate before entering a space to assess the potential risks. In this paper, we examine the potential of a novel yet cost-effective solution, called thermopile-based respiratory gating, to contactlessly screen a subject by measuring their respiratory rate in the scenario with an entrance gate. Based on a customized thermopile array system, we investigate different image and signal processing methods that measure respiratory rate from low-resolution thermal videos, where an automatic region-of-interest selection-based approach obtains a mean absolute error (MAE) of 0.8 breaths per minute. We show the feasibility of thermopile-based respiratory gating and quantify its limitations and boundary conditions in a benchmark (e.g., appearance of face mask, measurement distance and screening time). The technical validation provided by this study is helpful for designing and implementing a respiratory gating solution toward the prevention of the spread of COVID-19 during the pandemic.

## 1. Introduction

Coronavirus disease 2019 (COVID-19) is a novel coronavirus-induced respiratory disease, which has caused over 4,159,378 deaths as of 24 July 2021, according to [1]. It is urgent to take some measures to mitigate the outbreak of COVID-19. As difficulties in breathing is one of the major symptoms, the respiratory rate (RR) can be used as a critical physiological parameter to indicate the health deterioration or well-being of a person [2]. Since COVID-19 is a community-acquired pneumonia that is mainly transmitted through saliva droplets or discharge from the nose [3], people are therefore advised to wear face masks and have their body temperature measured at the entrance of public areas (e.g., train stations, airports, supermarkets, and libraries) to prevent the transmission of the virus [4]. However, body temperature only provides one dimension of information, and its accuracy is affected by environment (e.g., room temperature). The guidelines of National Institute for Health and Care Excellence (NICE) show that clinical features (e.g., body temperature ≥ 38 °C, respiratory rate ≥ 20 breaths per min, pulse rate > 100 min and crackles) could provide a rapid diagnosis of community-acquired pneumonia [5,6]. Adding one more variable (e.g., respiratory rate) can improve the accuracy of COVID-19 screening in public areas. Since COVID-19 is an infectious disease, we propose and examine the potential of a non-contact respiratory screening solution, called thermopile-based respiratory gating, that eliminates the risk of infection/contamination caused by sensing in a contact manner.

**Figure 1:**
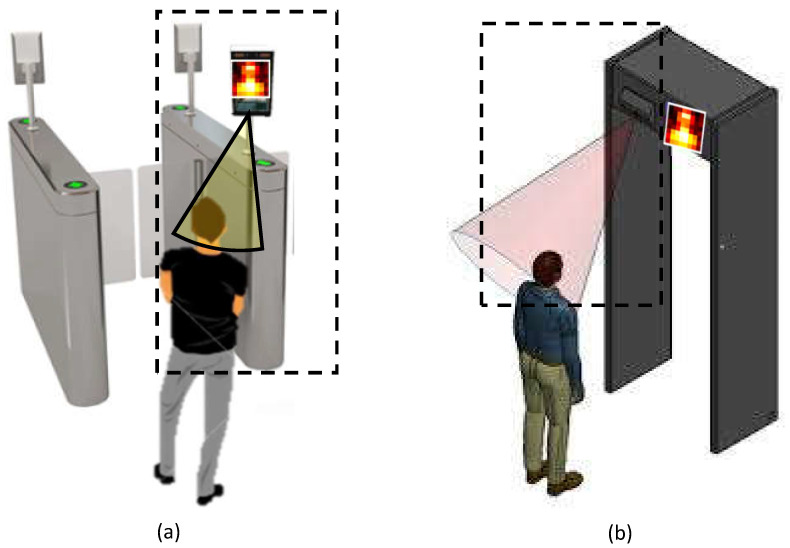
Illustration of the proposed thermopile-based respiratory gating solution that contactlessly screen the RR of a subject at the entrance gate. (**a**) Entry gate, (**b**) security gate.

The concept of respiratory gating is illustrated in Figure 1. Before entering a public space, subjects (with or without face mask) shall pass the gate, where a contactless sensor is used to measure and check the RR. In this application scenario, the sensor selection and algorithm design are critical. Since it aims at screening the RR of a subject in the stand position, the motion-based methods [7,8,9,10] and photoplethysmography-based methods [11,12,13] are not suitable for this scenario. The reasons are as follows: (i) it is difficult to accurately measure the chest/belly movement (induced by inhaling and exhaling) from a subject in the stand position due to involuntary body motions; (ii) it is difficult to detect the skin pulsation from limited skin areas under a face mask; (iii) the uncertainty and variation of the on-site illumination condition pose an extra challenge (i.e., unknown factor) for the measurement that requires an active light source (e.g., RGB or near-infrared camera); and (iv) motion-based respiratory rate measurements do not resemble the true measurement of nostril airflow. Therefore, we consider a thermography-based modality as an appropriate option here.

Thermal-based respiration monitoring has been proposed and demonstrated on both high and low resolution thermal cameras [14,15,16,17,18,19,20]. Based on our targeting scenario (i.e., a subject that may wear a face mask in the stand position at the entrance gate), we consider the low-resolution thermal camera as a feasible option, as the respiratory region of interest (ROI) is significantly decreased, due to the use of a face mask. Another consideration is that high-resolution thermal cameras are rather expensive such that they cannot be widely deployed in cost-sensitive areas. Therefore, we propose to use the low-cost thermopile array sensor to build the respiratory gating setup. The thermopile array sensor is comprised of a series of thermocouples, which detects the infrared radiation emitted by all objects within a certain temperature range. It has been widely used for human occupancy detection [21,22,23]. It is favored in contactless health monitoring applications because the thermopile array sensor evades privacy issues by its low-resolution property (e.g., 8×8 pixels), e.g., seizure detection during sleep [24], fall detection [25,26], sleep posture classification [27,28], household activities monitoring [29], bed-exit detection [30] and head/body posture detection [31].

Though low resolution is an attractive property for thermopile array sensors in terms of privacy protection, it remains a main challenge for image and video processing. It is impossible to perform face detection or facial landmark detection [14,15] on a thermopile image. Different approaches have been proposed to address the issue of ROI detection. Pereira [16] computed the signal quality index (SQI) of each ROI and selected suitable ROIs to extract the respiratory signal based on SQI values. Since the ROI to be selected contains breathing-induced motion and respiratory flow, the extracted signal is unreliable, especially when apnea events are present. Lorato [17] used nostril temperature changes to define the ROI, but since the nostril is a very small area, the distance between the subject and sensor needs to be very close (e.g., 10 cm). The method used in [17] is not suitable for our gating scenario, because it is difficult to require people to keep a certain distance from the sensor in public. To detect the RR from low-resolution thermal videos in our gating scenario, we built a setup in the lab and investigated different image processing and signal extraction approaches, which include the following: (i) full video processing that uses very simple spatial statistics of 8×8 pixels to generate the respiratory signal; (ii) ROI-base processing that performs a rough segmentation of respiratory and non-respiratory regions to refine the extraction of the respiratory signal. The feasibility of thermopile-based respiratory gating was demonstrated and its limitations and boundary conditions that may appear in real applications were fairly discussed. Fast Fourier transform (FFT) and inter-beat-intervals (IBI) are commonly used for respiratory rate calculation (averaged and instantaneous rates) [32]. However, the difference of these two respiratory rate calculations has not been thoroughly explored. We evaluated their differences in this work in the context of fast respiratory screening.

The main contribution of this paper is that we propose a novel concept for contactless respiratory gating that uses a cost-effective thermopile sensor and a simple image- and signal-processing method to screen the RR of a subject at the entrance of public areas, where the subject is in the standing position (with or without face mask). It targets a new application scenario in COVID-19 that helps in controlling the spread of the pandemic, using contactless health sensing technology. The proposed respiratory gating is cost effective and easy to deploy in practice. For image-processing algorithms, we present different options where an automatic ROI selection-based method is highlighted in our benchmark. We also report the limitations and boundary conditions of this proposal by quantifying the effect of the factors included (e.g., with/without face mask measurement distance and screening time). The obtained insights improve the understanding toward real applications. The remainder of this paper is organized as follows. In Section 2, we introduce the measurement setup for respiratory gating. In Section 3, we present and analyze six benchmark methods based on either the full video processing or ROI selection. Section 4 shows the experimental results and discussions. Finally, in Section 5, we draw the conclusions.

## 2. Setup and Measurement

This section introduces the measurement setup for thermopile-based respiratory gating, which was used to collect the benchmark dataset.

### 2.1. Experimental Setup

**Figure 2:**
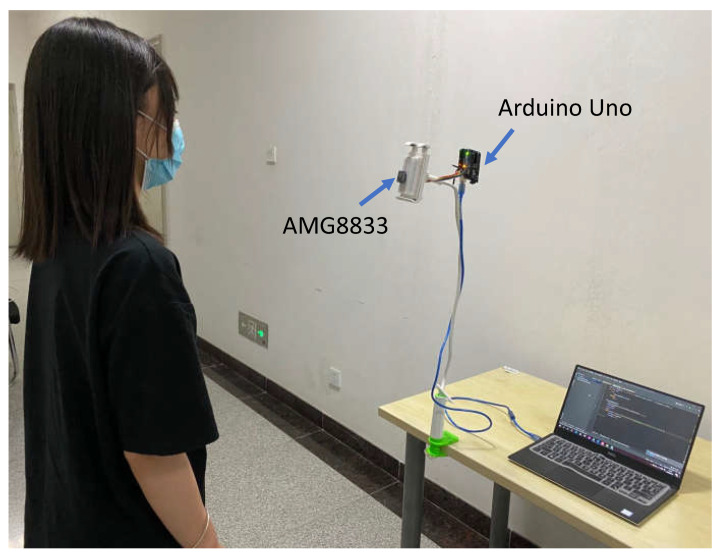
The experimental setup for recording the thermopile videos of a standing subject with a face mask.

To explore the feasibility of using a thermopile array sensor for respiratory gating, we built an experimental setup (see Figure 2) that consists of a Grid-Eye thermopile array AMG8833 from Panasonic, an Arduino Uno and a laptop. The thermopile sensor was placed in front of the subject with an angle of view of 60 degrees, roughly aiming at the nostril/mask area for nasal flow measurement. The thermal videos were recorded at a constant frame rate of 10 frames per second (fps). The spatial resolution is 8×8 pixels with absolute temperature distribution. During the recording, the thermopile array sensor was connected to an Arduino Uno, and the acquisition was performed through Python on a laptop with an Intel Core i5 processor (2.30 GHz). A moving mean filter was applied on the raw data of each video with a sliding window (with 1 s length) to reduce quantization noise.

### 2.2. Benchmark Dataset

A total of 75 videos were recorded from 12 healthy subjects (3 males and 9 females, aged from 22 to 68 years) with different configurations. Unless specified otherwise, each subject stood still in front of the thermopile array sensor and was guided to mimic the sinusoidal breathing pattern displayed on the frontal screen during the recording. The guided breathing signal had a duration of four minutes and the breathing frequency was changed from 10 to 30 breaths per minute (bpm). Specifically, the breathing frequency in the first and fourth minute was 20 bpm, and in the second and third minute, 10 bpm and 30 bpm, respectively. For the recording with a face mask, each subject was required to wear a surgical mask. This study was approved by Hunan University, and written informed consent was obtained from each subject.

#### 2.2.1. Dataset A: Guided Breathing with and without Face Mask

**Figure 3:**
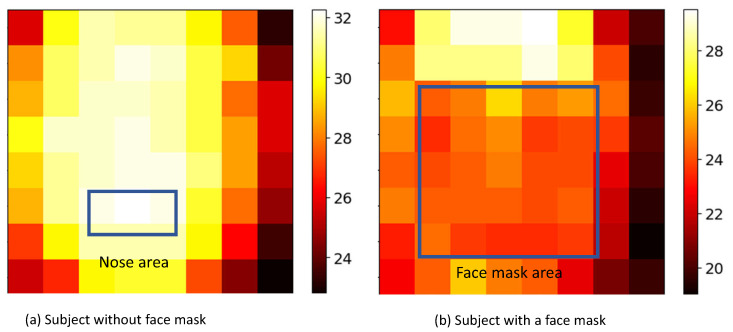
Examples of a subject face (with and without a face mask) captured by thermopile array sensor at the distance of 10 cm.

In real applications, the subject standing at the gate may not wear a face mask, and the distance between the subject and sensor may vary. We included these challenges in our experiments. Five scenarios with different subject-to-sensor distances were created, including the cases with and without a face mask. For the recordings with a face mask, the thermal videos were recorded at three different distances of 10, 30 and 50 cm. For the recordings without a face mask, we only performed recordings at distances of 5 cm (N-5 cm) and 10 cm (N-10 cm). Based on a pilot measurement, we found that the thermopile cannot measure the nostril temperature changes beyond the distance of 10 cm, due to the large quantization noise when the measurement is performed on the small nostril ROI with a few pixels [17]. Figure 3 exemplifies the image areas with respiratory flow induced heat exchange for a subject with and without a face mask. The waiting time of the subject at the gate is a critical factor that needs to be considered in practical applications. Therefore, we defined four different sliding window lengths of 5, 10, 20, and 30 s and analyzed the effects of different sliding window lengths to measure the respiratory rate on this dataset.

#### 2.2.2. Dataset B: Guided Breathing at Different Subject-to-Sensor Distances (with Face Mask)

To explore the boundary conditions for measurement distance (i.e., the maximum distance allowed between the sensor and subject for a valid measurement), the recordings were performed on a single subject with a face mask at multiple discrete distances, ranging from 10 to 150 cm with an interval of 10 cm.

## 3. Methods

**Figure 4:**
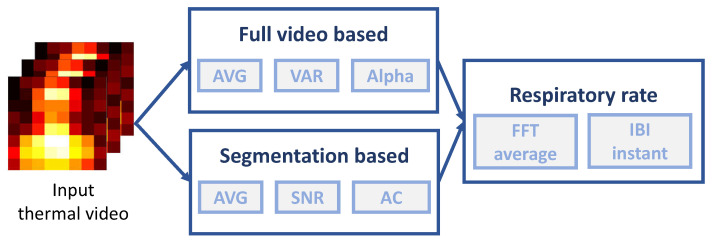
Overview of our algorithmic benchmark system. It consists of two image processing methods (full video based and segmentation based) for respiratory signal extraction and two methods for respiratory rate calculation. AVG—signal selection based on averaging; VAR—signal selection based on variation; Alpha—signal selection based on Alpha tuning; SNR—signal selection based on signal-to-noise ratio; AC—signal selection based standard deviation.

To extract the RR from low-resolution videos acquired by the thermopile array sensor, we explored different image processing approaches for respiratory signal extraction and different methods for respiratory rate calculation (see the overview in Figure 4). The image and signal processing methods are detailed in this section.

### 3.1. Full Video Processing-Based Methods (FVP)

In this subsection, we introduce three full video processing-based methods that use simple image statistics to create a respiratory signal.

#### 3.1.1. Averaging

Given a thermal video, we use I(x,y,t) to denote the temperature of a pixel at location (x,y) of a *t*-th thermal image. When the subject wears a face mask or the distance between the subject and sensor is very close (e.g., 5 cm), the respiratory region has a relatively large area in the thermal image. Therefore, we can temporally concatenate the spatially averaged pixel values to approximate a time signal that includes respiratory rhythm, denoted as AVG. For a thermal image with height *H*, and width *W*, the *t*-th respiratory signal AVG(t) calculated by AVG can be expressed as follows:

(1) $$AVG\left(t\right)=\frac{1}{HW}\sum_{x=1}^{H}\sum_{y=1}^{W}I\left(x,y,t\right)$$

#### 3.1.2. Variation

**Figure 5:**
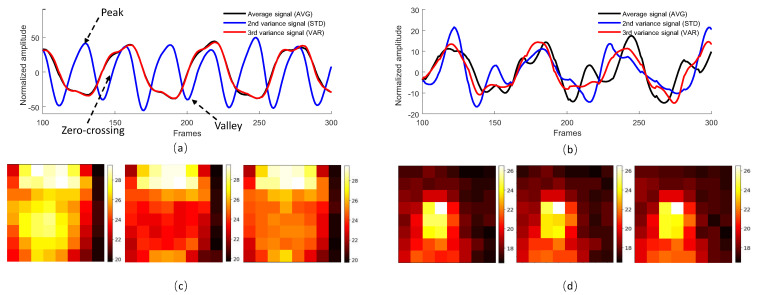
The spatially averaged, 2nd-order variation signal (standard deviation) and 3rd-order variation signal extracted from the thermopile video where the subject wears a face mask and the distance between the subject and sensor is (**a**) 10 cm and (**b**) 50 cm, respectively. The left column, middle column and right column of thermal images (**c**) are taken at the peak (exhaling), valley (inhaling) and zero-crossing of the respiratory signal (**a**). The left column, middle column and right column of thermal images (**d**) are taken at the peak (exhaling), valley (inhaling) and zero-crossing of the respiratory signal (**b**).

When the subject does not wear a face mask or the subject-to-sensor distance is large (e.g., 50 cm), the respiratory region will be small in the image. Hence, the spatially averaged signal resembles temperature variations of non-respiratory areas rather than the respiratory flow. According to [33], spatial mean and spatial standard deviation have a complementary effect in qualifying pixels. So, we use the spatial standard deviation of pixel values as an alternative to generate a time signal, denoted as VAR, i.e., the mean of the non-respiratory area is removed from the standard deviation representation. Since the standard deviation is calculated on the second order statistics, it does not reflect the polarity of the signal (e.g., values are all positive). To preserve the inhaling and exhaling phases in the standard deviation signal, we use the third power instead of the second power to compute the spatial variance. A comparison of the spatially averaged signal, 2nd-order and 3rd-order variation signals is shown in Figure 5. The *t*-th respiratory signal VAR(t) calculated by VAR can be expressed as follows:

(2) $$VAR\left(t\right)=\sqrt[{3}]{\frac{\sum_{x=1}^{H}\sum_{y=1}^{W}{\left(I\left(x,y,t\right)-\mu \right)}^{3}}{HW}}$$

where μ denotes the spatially averaged pixel values in the thermal image.

#### 3.1.3. Alpha Tuning

As described above, AVG and VAR have complementary temporal behaviors, i.e., if respiratory modulation is stronger in one signal, it will be weaker in another signal. Therefore, as the third approach, we propose to combine the AVG and VAR signals such that the respiratory component could be enhanced. In addition, the dependency on the respiratory area size will be lessened in a combined version. Similar to [34,35], we use alpha-tuning to combine the two signals with a positive sign in between (i.e., additive relationship). The rationale is that during exhaling, both the averaged value of the respiratory area (AVG) and the contrast between respiratory and non-respiratory areas (VAR) increase simultaneously, and vice versa for inhaling (see examples in Figure 5). Thus, the respiratory components in AVG and VAR signals should be in-phase, so adding two signals shall boost the strength of respiration. Therefore, the *t*-th respiratory signal Alpha(t) calculated by alpha tuning can be expressed as follows:

(3) $$Alpha\left(t\right)=AVG\left(t\right)+\frac{\sigma (AVG(t))}{\sigma (VAR(t))}\cdot VAR\left(t\right)$$

where σ denotes the standard deviation operator. Note that the AVG and VAR signals are centered to zero (i.e., zero-mean) before the alpha-tuning combination.

### 3.2. Segmentation-Based Methods (Seg)

**Figure 6:**
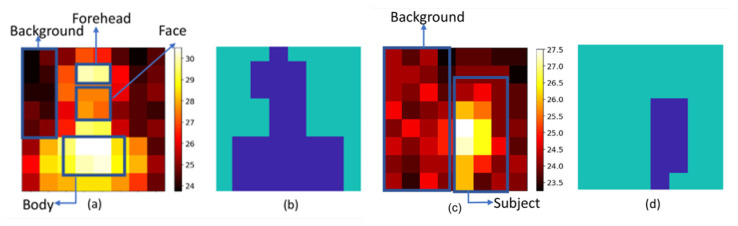
(**a**,**c**) Thermal images of subject with a face mask at the distance of 30 cm and 150 cm away from the sensor, respectively. (**b**,**d**) Automatically segmented foreground (navy blue) and background (green) regions by K-means clustering.

As the respiratory signal is extracted from low-resolution thermal images, facial landmark-based ROI detection cannot be applied. In our application scenario, the temperature of the background can be assumed to be lower than the temperature of the human body (as shown in Figure 6). Thus, we can separate the thermal image into foreground and background areas based on the DC-temperatures, where the DC-temperatures refer to temporally averaged temperature values in a time interval. First, we calculate the DC-temperature of each pixel in a thermal image sequence. Next, K-means clustering [36,37] is applied to these DC-temperature features to cluster the pixels into two groups, denoted as the foreground and background (the maximum and minimum DC-features are used as the initial centroids of K-means clustering, and the distance from each centroid to pixels is computed by squared Euclidean distance.) After that, we calculate the mean of each cluster and choose the one with the higher temperature as the foreground. We note that the foreground/background clustering is updated in a sliding window process in real-time, which will be introduced later.

#### 3.2.1. Averaging

Given the foreground that includes the respiratory area (face mask or nostril), we propose to use the spatially averaged pixel values of the foreground and concatenate them into a temporal trace in the similar way as AVG used for full video processing.

#### 3.2.2. SNR

To further exclude outliers from the foreground, such as forehead, neck and body (see Figure 6), we use the signal-to-noise ratio (SNR) as the quality metric to assess the quality of thermal signals measured from foreground pixels and select the ones with stronger respiratory energy as the output. The SNR is calculated as a ratio of the inband (e.g., [10, 50] bpm) and outband energies of the signal. Finally, the selected pixels are averaged in the temporal domain. Specifically, the SNR is calculated in the same sliding window used for K-means clustering.

#### 3.2.3. AC

In addition to SNR, another quality metric to select the respiratory regions is by AC, which refers to the standard variations of temperature values in the sliding window [18]. We compute the standard deviation of each pixel in the foreground and select the one with the highest standard deviation as the respiratory region.

### 3.3. Respiratory Rate Calculation

For all benchmarked respiratory signal extraction methods, a sliding window based process is applied in the time domain to measure and overlap/add the signals in shorter time intervals. Since different sliding window lengths mean different time latency for respiratory signal generation, we define four sliding window lengths (e.g., 5, 10, 20 and 30 s) to extract the respiratory signal. To suppress distortions, a bandpass filter with a low cut-off frequency of 0.167 Hz and a high cut-off frequency of 0.833 Hz is applied in the sliding window to eliminate signal components outside the respiratory band.

We investigated two different methods for the respiratory rate calculation (averaged rate and instantaneous rate). For each measurement, we have different evaluation metrics to assess its performance.

#### 3.3.1. Averaged Respiratory Rate

It is calculated in the frequency domain by taking the frequency index of the maximum spectrum peak within the respiratory band ([10,50] bpm) [13,35]. The frequency spectrum is derived within a short time interval by a sliding window (with 10 s length and 0.1 s sliding step). The averaged respiratory rates estimated in the sliding window are concatenated into a long rate trace.

We use mean absolute error (MAE) to measure the difference of averaged respiratory rates obtained by the thermopile array sensor and reference, Pearson correlation coefficient to evaluate their correspondence, and coverage to evaluate the percentage of correctly measured rates with an absolute error smaller than 3 bpm. MAE is defined as follows:

(4) $$MAE=\frac{\sum_{i=1}^{N}\left|RR_{pre}\left(i\right)-RR_{ref}\left(i\right)\right|}{N}$$

where $RR_{pre}$ and $RR_{ref}$ indicate the RR extracted from the thermopile array sensor and reference RR signal, respectively. The Pearson correlation coefficient is defined as follows:

(5) $$Pearson=\frac{\sum_{i=1}^{N}\left(RR_{pre}\left(i\right)-\bar{RR_{pre}}\right)\left(RR_{ref}\left(i\right)-\bar{RR_{ref}}\right)}{\sqrt[{2}]{\sum_{i=1}^{N}{\left(RR_{pre}\left(i\right)-\bar{RR_{pre}}\right)}^{2}\sum_{i=1}^{N}{\left(RR_{ref}\left(i\right)-\bar{RR_{ref}}\right)}^{2}}}$$

where $\bar{RR_{pre}}$ and $\bar{RR_{ref}}$ are the mean values of RR estimated from the thermopile array sensor and reference RR signal, respectively. Coverage is defined as follows:

(6) $$Coverage=\frac{C}{N}$$

where *C* represents the number of $RR_{pre}\left(i\right)$ in the range of $\left[RR_{ref}\left(i\right)-3,RR_{ref}\left(i\right)+3\right]$.

#### 3.3.2. Instantaneous Respiratory Rate

It is derived in the time domain by taking the inverse of inter-breaths-intervals between the detected respiratory peaks (due to inhaling) [12,38], which is, therefore, more sensitive to spontaneous respiratory changes. To quantify the beat-to-beat accuracy of the measurement, we use the following two metrics to assess the detected respiratory peaks: (i) precision, which denotes the percentage of valid camera measurement w.r.t. the total number of detected camera peaks (e.g., accuracy); and (ii) recall, which denotes the percentage of valid camera measurement w.r.t. the total number of reference peaks (e.g., sensitivity or retrieval rate). We define the *i*-th respiratory peak detected by the thermopile array sensor as $P_{pre}\left(i\right)$ and that detected by the reference RR signal as $P_{ref}\left(i\right)$. If there is only one $P_{pre}\left(i\right)$ in the range of $0.5*[P_{ref}\left(i-1\right)+P_{ref}\left(i\right),P_{ref}\left(i\right)+P_{ref}\left(i+1\right)]$, the $P_{pre}\left(i\right)$ is a valid peak measured by the sensor. The precision and recall are defined as follows:

(7) $$precision=\frac{NP_{valid}}{NP_{pre}}$$

(8) $$recall=\frac{NP_{valid}}{NP_{ref}}$$

where $NP_{pre}$ and $NP_{ref}$ represent the number of respiratory peaks detected by the thermopile array sensor and reference RR signal, and $NP_{valid}$ indicates the number of valid peaks detected by the thermopile array sensor.

## 4. Results and Discussion

In this section, we first report the benchmark results of respiratory signal extraction methods on Dataset A and discuss the feasibility of thermopile-based respiratory gating. Next, we discuss the robustness and sensitivity of processing with different sliding window lengths (time latency) and compare the performance of averaged and instantaneous respiratory rates for this application. Finally, we investigate the distance range allowed for measurement on Dataset B.

### 4.1. Feasibility of Thermopile-Based Respiratory Gating

Table 1 shows the averaged metric values obtained by six benchmarked methods, from which we can see that all methods perform better in the category where subjects wear a face mask than that without a face mask. This is expected, as face masks increase the spatial area with respiration-induced temperature changes, against the nasal respiration measurement, where only the nostril temperature changes can be measured. At the same sensor distance, the area that can be used for respiration measurement is significantly increased by a face mask. Moreover, the larger respiratory area allows subject to stand in a less restricted direction w.r.t. the aiming angle of the thermopile array sensor. For nasal measurement, the viewing angle of the sensor is more demanding and critical, as it needs to “see” the temperature changes of the small nostril area (i.e., bottom-up angle is recommended in the stand position). Regarding the feasibility of this measurement, a high-level conclusion is the following: for this measurement (with this dataset), the best measurement coverage and MAE we obtained for the scenario without face mask are 73.4% and 4.8 bpm, and for the scenario with a face mask, 96.2% and 0.8 bpm.

From Table 1, we also conclude that Seg-based methods are generally better than FVP-based methods. Their major difference is in the scenario without a face mask, as Seg-based methods can more accurately locate the small respiratory area and exclude the background. In view of the results obtained in the scenario with a face mask, we feel that the differences between benchmarked methods are not significant, which means that simple image statistics based methods can attain generally good performance in this use case (especially during the COVID-19 period, where subjects are demanded to wear face mask).

**Figure 7:**
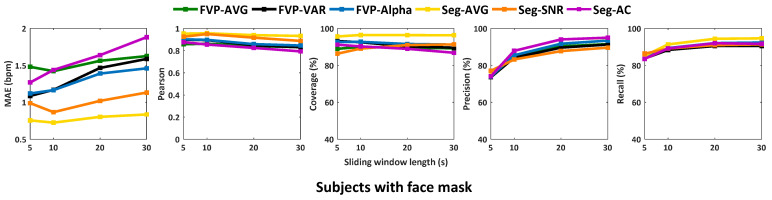
The performance curves of six benchmarked methods in the scenario with a face mask. The curves are obtained with different sliding lengths for verifying the reproducibility of conclusions with different time latencies for processing and the sensitivities of different methods to the time window.

A more detailed analysis is shown in Figure 7 that focused on the comparison in the scenario with a face mask (the targeted COVID-19 use case of this study). It confirms the conclusions we have drawn from Table 1: Seg-based methods have generally better performance than FVP-based methods, and this conclusion is consistent with different sliding window lengths (latency). As explained, this is due to the advantage of foreground and background separation of Seg, i.e., see the comparison between FVG-AVG and Seg-AVG, where both use the same method (spatial averaging) to create a respiratory signal. However, we emphasize an intrinsic limitation of image segmentation for thermopile array sensors: fine-grained segmentation/separation of objects is not possible in the low-resolution image (8×8 pixels). Additionally, Figure 7 shows that FVP-Alpha slightly improves the performance of FVP-AVG and FVP-VAR, suggesting that the combination of two in-phase signals can indeed enhance the respiratory energy, compared to their separate versions.

### 4.2. Analysis of Processing Time Latency (Sliding Window Lengths)

As this study aims for the application of “vital signs screening”, the processing latency (defined as sliding window length) is a critical parameter to be investigated. Longer sliding window length will certainly improve the measurement robustness and stability, as it includes more respiratory circles, but it also increases the waiting time for the first measurement, which is less appreciated in terms of user experience (i.e., subjects need to wait longer at the entrance).

Table 2 summarizes the statistical values of Figure 7 with a focused discussion on Seg-based methods. Seg-AVG has the overall best performance in this evaluation, i.e., fewer variations in different sliding window lengths. The reason is that Seg-AVG uses simple spatially averaged values rather than SNR or AC properties that rely on temporal characteristics of the signal. In comparison, Seg-SNR and Seg-AC use temporal properties of the signal to make the selection, which is more sensitive to the sliding window length. In the case of a short sliding window, the frequency resolution of respiratory components is low and the differentiation between respiratory and non-respiratory components will be more difficult. If the sliding window contains significant respiratory-rate changes (i.e., from 20 bpm to 10 bpm in our protocol), the respiratory frequency spectrum will be more spread (less spiky) and the SNR will be lower in our definition, which may lead to wrong ROI selection for Seg-SNR. The same holds for Seg-AC. The major difference between Seg-SNR and Seg-AC is that the AC selection is not total temporal energy normalized, which might be more sensitive to sensor noise or motion disturbance/trend with a frequency lower than the respiratory signal. However, if the sliding window is too short (e.g., 5 s or even shorter), such normalization will be unstable.

Considering the measurement performance and user experience (waiting time) for respiratory gating, we recommend Seg-AVG for this application, with a processing latency (sliding window length) of 10 s.

### 4.3. Analysis of Methods for Respiratory Rate Calculation

**Figure 8:**
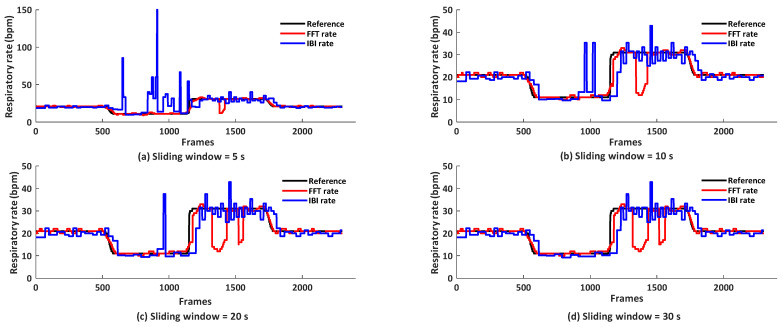
FFT-based and IBI-based respiratory rates obtained by FVP-AVG under different sliding window lengths on Dataset A, where subject wears a face mask at the sensor distance of 30 cm.

Figure 7 and Table 2 show that the averaged RR and instantaneous RR have opposite sensitivity to the sliding window length. The performance of the averaged rate decreases with the increase of the sliding window length, but this is the other way around for the instantaneous rate. We probe the reasons: this is due to the different ways of rate calculation. The averaged rate is calculated in the frequency domain. The selection of respiratory component might be confused if larger lower frequency components/trends are included in the window, such as involuntary body motion, as is typical in the standing position, yet motion tracking and compensation are not ideal in the low-resolution video. Conversely, instantaneous RR obtained by inter-beat-intervals between inhaling peaks detected in the time domain is more sensitive to high-frequency jitters/noise in short windows. The inhaling peak detection exploits the waveform characteristics like morphology, which is less visible in short window intervals. We conclude that for shorter sliding window lengths (e.g., 5 s), an averaged rate is preferred; for longer sliding window lengths (e.g., 30 s) where the waveform morphology is clearer, a peak-to-peak based instantaneous rate is preferred. For visual comparison, we show the examples of averaged and instantaneous rates obtained by FVP-AVG under different window lengths in Figure 8.

### 4.4. Distance Range for Respiratory Gating

**Figure 9:**
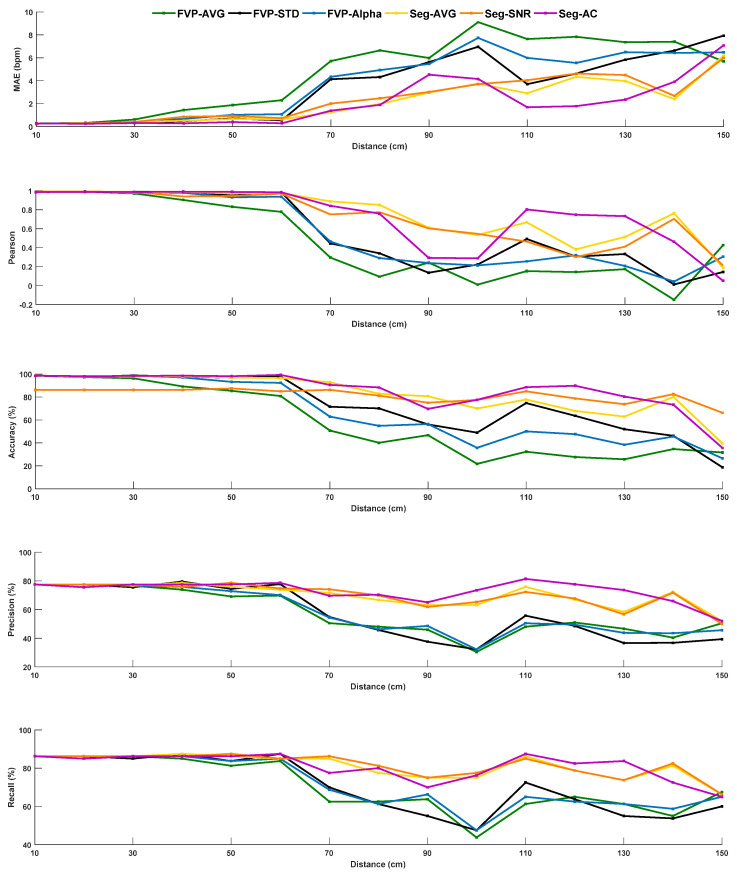
Statistical results obtained by six benchmarked methods on the Dataset B where subject wears a face mask under different sensor distances varying from 10 cm to 150 cm with an interval of 10 cm.

As mentioned before, as respiratory gating should provide a short screening time with less processing latency, we define the default sliding window length as 5 s (50 frames at 10 fps) for this experiment (on Dataset B). Figure 9 suggests that FVP-based methods are more sensitive to the distance between the sensor and subject, which is in line with our expectation. The subject’s body parts become substantially smaller in the thermopile image with the increase in measurement distance, i.e., it is not possible to differentiate different body parts at the maximum distance of 150 cm in this experiment. Seg-based methods also suffer from degradation with the increase in distance, but their quality drops are less significant than FVP-based methods, due to the foreground and background separation (by K-means). This indicates that a basic/simple regional segmentation in a 8×8 pixels image is still helpful for improving the robustness to the variations/uncertainties of measurement distance. However, we note that when the distance is larger than 60 cm, Seg-based methods do not perform well, i.e., at this distance, it is already difficult to separate different facial parts. Based on the setup and pilot measurement built in the lab, we suggest the distance range for this application (i.e., respiratory screening in the stand position) to be less than 60 cm.

## 5. Conclusions

This paper examined the potential of a novel application for the timely and important research topic of COVID-19 controlling: contactless screening of RR at the entrance gate by a low-cost thermopile array sensor. Based on a setup we built in the lab, we explored different image and signal processing methods to extract the RR from challenging low-resolution thermal images, i.e., full-video-based or segmentation-based methods, as core algorithms to extract the respiratory signals. In the benchmark based on two datasets, we demonstrated the feasibility of thermopile-based respiratory gating, and analyzed the sensitivity of such an application in view of realistic challenges, such as with/without a face mask, measurement distance, and screening time. We compared different options of image processing and highlighted a simple solution based on the rough segmentation of the respiratory area. We also concluded the merits and drawbacks of different ways to calculate respiratory rates (averaged or instantaneous) in different time window lengths, and the distance range for measurement. Due to the current restrictions on conducting clinical experiments on COVID patients, the proposed solution was not validated in a clinical trial with COVID-19 patients; the proposal is a technical proof-of-concept. Future research should focus on validation with COVID-19 patients. The insights provided by this study may initiate further exploration and development of the novel concept of contactless respiratory gating, toward multi-modal physiological sensing, including respiration and skin temperature, to combat COVID-19.

## Figures and Tables

**Table 1:** Statistical results obtained by six benchmarked methods on Dataset A, using the default setting. **Boldface** character denotes the best result per row.

Metric	Mask	FVP			Seg		
		AVG	VAR	Alpha	AVG	SNR	AC
MAE (bpm)	N	5.1	5.3	5.1	4.9	**4.8**	5.1
	Y	1.5	1.3	1.3	**0.8**	1.0	1.6
Pearson	N	0.33	0.29	0.32	**0.36**	0.32	**0.36**
	Y	0.85	0.87	0.88	**0.95**	0.92	0.84
Coverage (%)	N	49.5	46.6	48.8	53.2	**73.4**	48.2
	Y	89.5	91.4	92.1	**96.2**	89.5	89.4
Precision (%)	N	**71.4**	68.5	68.9	71.3	71.2	70.1
	Y	85.3	84.7	86.2	**87.8**	84.4	**87.8**
Recall (%)	N	72.50	69.9	71.3	**73.2**	73.0	70.2
	Y	88.9	88.3	89.5	**91.4**	89.5	89.1

**Table 2:** Statistical results obtained by Seg-based methods on Dataset A, where subjects wear a face mask. **Boldface** character denotes the best result per row.

Metric	L = 5 s			L = 10 s			L = 20 s			L = 30 s		
	AVG	SNR	AC	AVG	SNR	AC	AVG	SNR	AC	AVG	SNR	AC
MAE (bpm)	**0.76**	0.99	1.27	**0.73**	0.87	1.44	**0.80**	1.02	1.64	**0.84**	1.13	1.88
Pearson	**0.95**	0.93	0.88	**0.96**	0.95	0.86	**0.94**	0.92	0.82	**0.93**	0.89	0.79
Coverage (%)	**95.7**	86.4	91.2	**96.5**	89.1	90.3	**96.4**	91.0	89.0	**96.4**	91.4	86.9
Precision (%)	74.9	**77.1**	74.2	87.8	83.2	**87.9**	**94.0**	87.8	**94.0**	94.7	89.6	**95.0**
Recall (%)	85.2	**86.4**	83.6	**91.4**	89.1	89.2	**94.4**	91.0	92.0	**94.7**	91.4	91.7

## Data Availability

Not applicable.
